# Abdominal Pain and Hyperlactatemia in Thiamine Deficiency: A Case Report

**DOI:** 10.1155/crpe/4401612

**Published:** 2026-06-26

**Authors:** Luciana Melo Campos, Roberto José Negrão Nogueira, Mariana Tresoldi das Neves Romaneli, Maria Giovana Oliveira Farias, Joaquim Bustorff-Silva

**Affiliations:** ^1^ Pediatric Department, FCM-UNICAMP, Campinas, Sao Paulo, Brazil, unicamp.br; ^2^ Medical Clinic Department, FCM-UNICAMP, Campinas, Sao Paulo, Brazil, unicamp.br; ^3^ Surgery Department, FCM-UNICAMP, Campinas, Sao Paulo, Brazil, unicamp.br

**Keywords:** abdominal pain, beriberi, hyperlactatemia, thiamine deficiency

## Abstract

An eight‐year‐old boy with recurrent abdominal pain, refusal to eat, gagging, and weight loss for five months experienced a sudden clinical deterioration after a gastrointestinal endoscopy with biopsy. Initially misdiagnosed with pancreatitis, he underwent hospitalization, fasting, and parenteral nutrition (PN) for 15 days. Despite this, his symptoms persisted, and serum lactate levels progressively increased. Transferred to a university hospital, abdominal beriberi was suspected, and intravenous thiamine was promptly administered. Symptoms resolved within 24 h. A review of the PN prescribed revealed the absence of thiamine, confirming the diagnosis. Healthcare professionals often associate thiamine deficiency with alcoholic adults presenting Wernicke encephalopathy; however, abdominal beriberi should also be considered in children with acute abdomen, especially when elevated serum lactate indicates impaired cellular metabolism due to thiamine deficiency.

## 1. Introduction

Beriberi, a disease caused by vitamin B1 (thiamine) deficiency, can lead to a range of metabolic, neurological, cardiovascular, respiratory, gastrointestinal, and muscular disturbance. The acute form is usually life‐threatening and typically manifests as Wernicke encephalopathy or as wet beriberi, particularly in patients with chronic alcohol misuse^,^ [1, 2]. Abdominal beriberi is the rarest acute manifestation of the disease and is attributed to dysfunction of the myenteric plexus, resulting in severe abdominal pain, nausea, vomiting, anorexia, constipation, and diarrhea. In some cases, it mimics an acute surgical abdomen [3, 4].

Once beriberi is suspected, prompt thiamine administration is essential to prevent the morbidity and mortality associated with its acute form. In such cases, intravenous thiamine infusion leads to rapid and near‐complete resolution of the symptoms [2, 3, 5]. This case report aims to reinforce the importance of considering beriberi as a potential diagnosis in patients with abdominal pain and hyperlactatemia.

## 2. Case Report

An eight‐year‐old boy was being followed in an outpatient setting due to intermittent and recurrent abdominal pain, refusal to solid foods, gagging, and significant weight loss (16%) over a five‐month period. During this time, several potential diagnoses were ruled out, including psychiatric disorders, IgA deficiency, coeliac disease, inflammatory bowel disease, and eosinophilic esophagitis. To further investigate the symptoms, a gastrointestinal endoscopy with collection of tissue samples was performed. Shortly afterward, the patient developed worsening abdominal pain and vomiting, prompting hospital admission.

**FIGURE 1:**
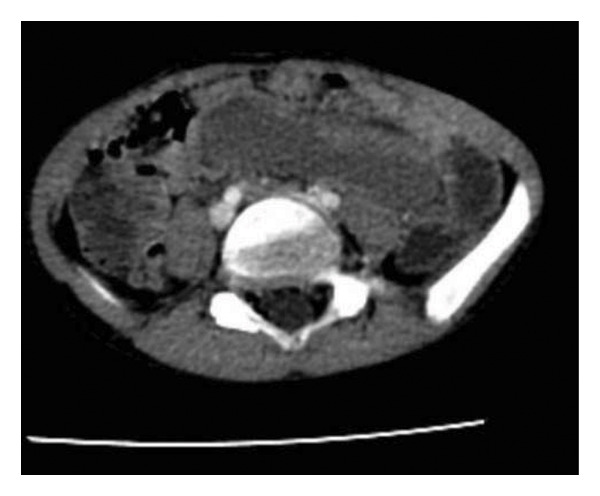
Duodenal hematoma.

Laboratorial tests revealed elevated amylase (538 U/L, normal range 22–80) and lipase (767 U/L, normal range < 67), suggestive of acute pancreatitis. A computed tomography (CT) scan was performed to evaluate the pancreas; however, instead of pancreatic abnormalities, the scan showed a moderate duodenal hematoma, measuring approximately 25 cm (Figure 1), probably caused by the biopsy and resulting in bowel obstruction. All previously considered diagnoses were definitively excluded during this hospitalization.

Over the next 15 days, the patient remained fasting and received total parenteral nutrition (PN), which led to a reduction in vomiting frequency and a gradual decline in values of amylase and lipase levels. Despite these improvements, the patient’s abdominal pain progressively worsened, and serum lactate levels continued to rise (Table 1). As the diagnosis remained uncertain, the child was transferred to a university hospital, where he was assessed by the nutritional multidisciplinary team (NMT), who raised the suspicion of abdominal beriberi. During the clinical course, the patient did not present alterations in mental status, ataxia, or ocular motility impairment, ruling out neurological presentations of beriberi.

**TABLE 1:** Laboratory evolution demonstrating a decrease in serum lactate.

	21/03/24	23/03/24	29/03/24	01/04/24	04/04/24	10/04/24	11/04/24–thiamine onset	15/04/24	Reference values
Amylase	538	445	367	336	318	289			22–80 U/L
Lipase	767	496	501			423			5–31 U/L
Serum lactate					5.4	11.5	9.4	1.8	0.5–1.6 mmol/L

Therefore, the treatment with intravenous infusion of thiamine (15 mg/kg/day for 5 days) was immediately prescribed, leading to pain relief within the first 24 h and to its disappearance within 48 h of infusion. Concurrently, serum lactate levels decreased from 11.5 to 1.8 mmol/L, and the patient resumed a solid diet without the recurrence of nausea, vomiting, or abdominal pain.

Although the patient reportedly had adequate thiamine intake in his regular diet before hospitalization, a review of the PN formulation administered at the previous hospital revealed the absence of thiamine, confirming the diagnosis of beriberi. A follow‐up CT scan showed resolution of the duodenal hematoma, and laboratorial analysis revealed normalization of pancreatic enzyme levels. The patient was discharged after completing a five‐day course of thiamine, with no recurrence of the symptoms.

## 3. Discussion

**FIGURE 2:**
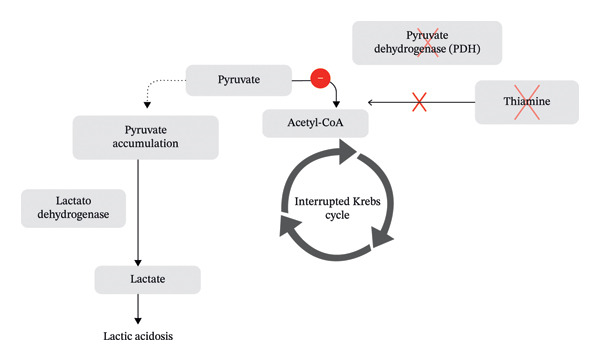
Mechanism of lactate increase in thiamine deficiency.

Thiamine plays a critical role in the Krebs cycle, as a component of thiamine pyrophosphate (TPP), a cofactor for enzymes pyruvate dehydrogenase and alpha‐ketoglutarate dehydrogenase. These enzymes catalyze the oxidative decarboxylation of pyruvate (from glycolysis) to acetyl‐CoA and of alpha‐ketoglutarate to succinyl‐CoA, both essential for ATP production [2]. In thiamine deficiency, the activity of these enzymes is impaired, reducing the conversion of pyruvate to acetyl‐CoA and leading to excess pyruvate, converted into lactate via anaerobic metabolism by lactate dehydrogenase (Cori cycle), ultimately contributing to hyperlactatemia (Figure 2) [6–8].

Besides thiamine, the Krebs cycle depends on adequate oxygen delivery and tissue perfusion. For this reason, most reports in the literature associate hyperlactatemia—indicative of impaired oxidative metabolism—with conditions such as shock and sepsis [9, 10]. However, in well‐oxygenated and hemodynamically stable patients, thiamine deficiency should not be overlooked as a potential cause.

This case illustrates a series of clinical challenges: an acute form of beriberi, typically seen in alcoholic adults, presented in a pediatric patient. Moreover, it did not follow the classic patterns of dry beriberi (Wernicke encephalopathy) or wet beriberi (cardiac failure) but instead manifested as abdominal beriberi—a rare and underrecognized variant (Table 2). The case was further complicated by the unreported absence of B‐complex vitamins, including thiamine, in the PN administered during 15 days of fasting [2, 11]. Additional confounding factors contributed to the diagnostic delay. The combination of abdominal pain, vomiting, and elevated amylase and lipase levels led to a presumptive diagnosis of acute pancreatitis, prompting fasting and PN. However, the lack of clinical improvement during the first week should have led to the reconsideration of the initial hypothesis. The duodenal hematoma, likely caused by endoscopic biopsy, was later identified and may have contributed to the symptoms and secondary pancreatitis by obstructing the pancreatic duct. Following resolution of the hematoma, persistent symptoms were initially misinterpreted as suggestive of a duodenal cyst. Ultimately, however, they were correctly attributed to abdominal beriberi, confirmed by the absence of thiamine in the PN formulation [12, 13]. Furthermore, gastrointestinal disorders, such as nausea, vomiting, abdominal pain, and diarrhea, are recognized contributors to deficiencies in B‐complex vitamins, especially thiamine, niacin, and folate [14].

**TABLE 2:** Comparison of Beriberi manifestations.

Type of beriberi	Affected system	Symptoms
Dry beriberi	Peripheral and central nervous system	Numbness in the extremities, muscle weakness, tingling, loss of reflexes, muscle pain, and difficulty walking. In severe cases, mental confusion, memory loss, and abnormal eye movements may occur.
Wet beriberi	Cardiovascular	Tachycardia, dyspnea on exertion and lying down, lower limb edema, heart failure, congestion, and hypotension
Abdominal beriberi	Gastrointestinal	Acute and intense or insidious abdominal pain, nausea, vomiting, anorexia, and hyperlactatemia.

Typically, mesenteric ischemia manifests with sudden, intense, and disproportionate abdominal pain, which was not observed in this case. Furthermore, at the time of our evaluation, the patient had been experiencing symptoms for several days, without signs of clinical worsening consistent with intestinal necrosis, which would be expected in untreated cases. Although CT angiography remains the diagnostic gold standard, it was not performed, as the subacute course and sustained clinical stability together lowered the probability of mesenteric ischemia. Therefore, while this diagnosis should be considered in cases of acute abdomen, the overall clinical context described here made it unlikely.

**FIGURE 3:**
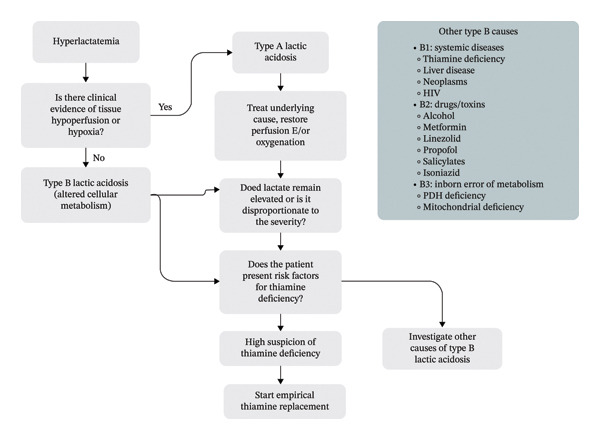
Diagram to be suspicious of thiamine deficiency.

In abdominal beriberi, pain is believed to result from altered perfusion of the myenteric plexus, producing severe pain akin to tissue ischemia [5, 15]. The present case met three key criteria for abdominal beriberi: hyperlactatemia in a well‐oxygenated and well‐perfused patient, dramatic symptom resolution following thiamine administration, and documented absence of thiamine PN administered for over 15 days. The first two criteria should alert health professionals to consider thiamine deficiency in cases of unexplained acute abdominal pain and hyperlactatemia (Figure 3). The third highlights a persistent issue in clinical practice: Despite clear guidelines, PN is still frequently prescribed without appropriate vitamin supplementation in many hospitals.

One potential limitation of this case report is the absence of serum thiamine measurement. Although laboratory confirmation can provide supportive evidence, it is not mandatory for the diagnosis in a typical clinical scenario. In fact, current literature emphasizes that, when thiamine deficiency is strongly suspected, treatment should be delayed pending laboratory results [16, 17]. Many institutions, including this university hospital, do not have access to timely thiamine assays, and their unavailability should not preclude appropriate clinical decision‐making.

In this case, the diagnosis of abdominal beriberi was supported by a constellation of findings: the presence of unexplained and progressively worsening abdominal pain, rising lactate levels despite adequate oxygenation and perfusion, documented absence of thiamine in the PN formulation, and, most importantly, a rapid and complete clinical response to thiamine administration. Although there was no report of clinical signs of vitamin deficiency prior to this hospitalization, it is impossible to rule out that previous loss of appetite contributed to the condition. However, the dramatic improvement within 24–48 h, accompanied by biochemical normalization, is both diagnostically and therapeutically specific for thiamine deficiency. In such cases, the therapeutic response itself becomes a reliable and sufficient diagnostic criterion. Recognizing and treating thiamine deficiency based on clinical judgment remains a crucial and life‐saving skill for healthcare providers worldwide, particularly in settings where access to timely laboratorial testing is limited.

## Author Contributions

Study design: Luciana Melo Campos, Roberto José Negrão Nogueira, and Mariana Tresoldi das Neves Romaneli.

Data collection: Luciana Melo Campos, Mariana Tresoldi das Neves Romaneli, Maria Giovana Oliveira Farias, and Joaquim Bustorff‐Silva.

Data analysis: Luciana Melo Campos, Roberto José Negrão Nogueira, Mariana Tresoldi das Neves Romaneli, and Joaquim Bustorff‐Silva.

Manuscript writing: Luciana Melo Campos, Roberto José Negrão Nogueira, and Mariana Tresoldi das Neves Romaneli.

Manuscript revision: Luciana Melo Campos, Roberto José Negrão Nogueira, and Mariana Tresoldi das Neves Romaneli.

Study supervision: Roberto José Negrão Nogueira and Mariana Tresoldi das Neves Romaneli.

## Funding

This study did not receive any funding.

## Conflicts of Interest

The authors declare no conflicts of interest.

## Supporting Information

Additional supporting information can be found online in the Supporting Information section.

## Supporting information


**Supporting Information** Supporting File 1: Serial laboratory data demonstrating the temporal evolution of serum lactate, amylase, and lipase levels during hospitalization. Supporting File 2: Summary table comparing clinical manifestations of dry, wet, and abdominal beriberi. Supporting File 3: Abdominal computed tomography image showing the duodenal hematoma. Supporting File 4: Diagram illustrating the mechanism of hyperlactatemia in thiamine deficiency. Supporting File 5: Clinical flowchart highlighting key features suggestive of thiamine deficiency in patients with abdominal pain and hyperlactatemia.

## Data Availability

The data supporting the findings of this study are available from the corresponding author upon reasonable request.
