# Supplementary material for: Abdominal Pain and Hyperlactatemia in Thiamine Deficiency: A Case Report
Source: Case Rep Pediatr. 2026 Jun 26;2026:4401612. doi: 10.1155/crpe/4401612 (PMC13308657; doi:10.1155/crpe/4401612)

**SUPPLEMENTARY MATERIALS**

**Supplementary File 1:** Serial laboratory data demonstrating the temporal evolution of serum lactate, amylase, and lipase levels during hospitalization.

**Supplementary File 2:** Summary table comparing clinical manifestations of dry, wet, and abdominal beriberi.

**Supplementary File 3:** Abdominal computed tomography image showing the duodenal hematoma.

**Supplementary File 4:** Diagram illustrating the mechanism of hyperlactatemia in thiamine deficiency.

**Supplementary File 5:** Clinical flowchart highlighting key features suggestive of thiamine deficiency in patients with abdominal pain and hyperlactatemia

Table 1 - Laboratory evolution demonstrating a decrease in serum lactate

|  | 21/03/24 | 23/03/24 | 29/03/24 | 01/04/24 | 04/04/24 | 10/04/24 | 11/04/24 - thiamine onset | 15/04/24 | Reference values |
| --- | --- | --- | --- | --- | --- | --- | --- | --- | --- |
| Amylase | 538 | 445 | 367 | 336 | 318 | 289 |  |  | 22-80 U/L |
| Lipase | 767 | 496 | 501 |  |  | 423 |  |  | 5-31 U/L |
| Serum lactate |  |  |  |  | 5,4 | 11,5 | 9,4 | 1,8 | 0,5-1,6 mmol/L |

Table 2 - Comparison of Beriberi manifestations

| **Type of Beriberi** | **Affected system** | **Symptoms** |
| --- | --- | --- |
| **Dry Beriberi** | Peripheral and central nervous system | Numbness in the extremities, muscle weakness, tingling, loss of reflexes, muscle pain, difficulty walking. In severe cases, mental confusion, memory loss, and abnormal eye movements may occur. |
| **Wet Beriberi** | Cardiovascular | Tachycardia, dyspnea on exertion and lying down, lower limb edema, heart failure, congestion, hypotension |
| **Abdominal Beriberi** | Gastrointestinal | Acute and intense or insidious abdominal pain, nausea, vomiting, anorexia, hyperlactatemia. |


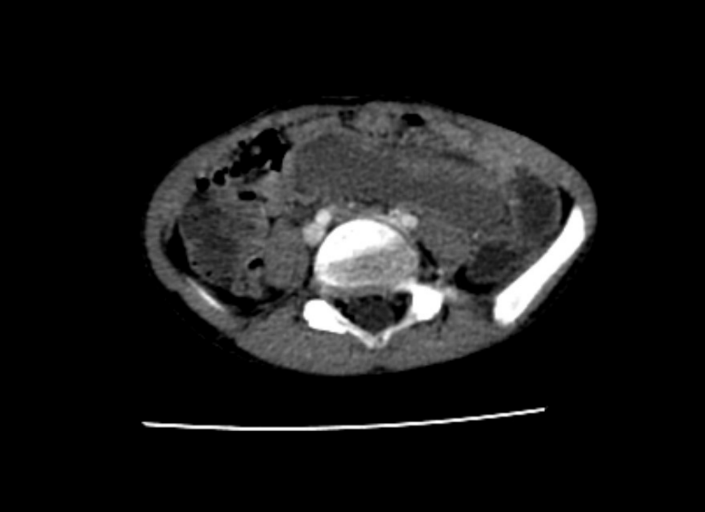


*Figure 1: duodenal hematoma*


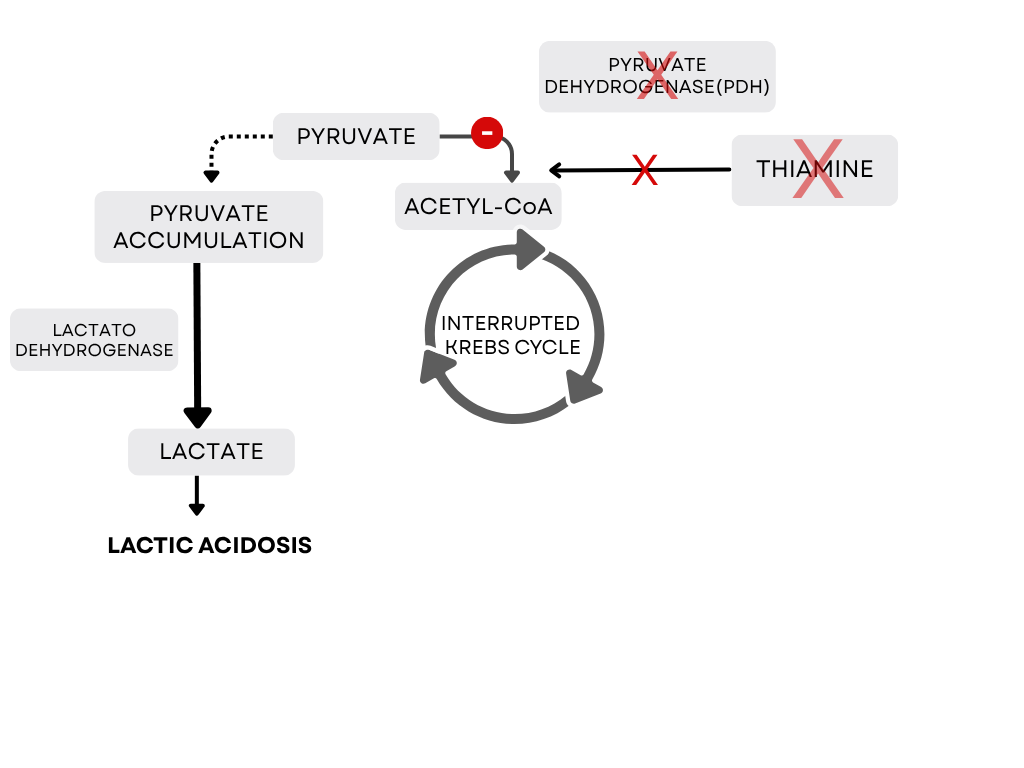


*Figure 2 - Mechanism of lactate increase in thiamine deficiency*

Figure 3 - Diagram to be suspicious of thiamine deficiency.
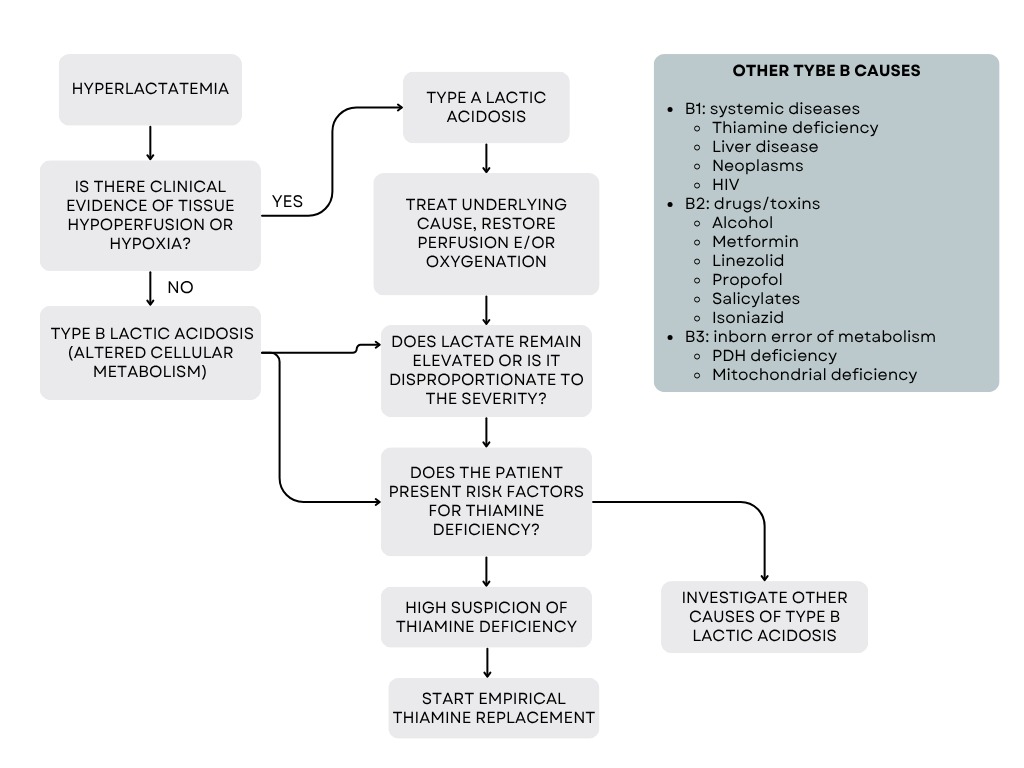

Supplement: Supplementary file 1 — Supporting Information Supporting File 1: Serial laboratory data demonstrating the temporal evolution of serum lactate, amylase, and lipase levels during hospitalization. Supporting File 2: Summary table comparing clinical manifestations of dry, wet, and abdominal beriberi. Supporting File 3: Abdominal computed tomography image showing the duodenal hematoma. Supporting File 4: Diagram illustrating the mechanism of hyperlactatemia in thiamine deficiency. Supporting File 5: Clinical flowchart highlighting key features suggestive of thiamine deficiency in patients with abdominal pain and hyperlactatemia. [file CRPE-2026-4401612-s001.zip › materiais suplementares.docx]
