# Supplementary material for: Abdominal Pain and Hyperlactatemia in Thiamine Deficiency: A Case Report
Source: Case Rep Pediatr. 2026 Jun 26;2026:4401612. doi: 10.1155/crpe/4401612 (PMC13308657; doi:10.1155/crpe/4401612)
Supplement: Supplementary file 1 — Supporting Information Supporting File 1: Serial laboratory data demonstrating the temporal evolution of serum lactate, amylase, and lipase levels during hospitalization. Supporting File 2: Summary table comparing clinical manifestations of dry, wet, and abdominal beriberi. Supporting File 3: Abdominal computed tomography image showing the duodenal hematoma. Supporting File 4: Diagram illustrating the mechanism of hyperlactatemia in thiamine deficiency. Supporting File 5: Clinical flowchart highlighting key features suggestive of thiamine deficiency in patients with abdominal pain and hyperlactatemia. [file CRPE-2026-4401612-s001.zip › materiais suplementares.docx.pdf]

SUPPLEMENTARY MATERIALS

Supplementary File 1: Serial laboratory data demonstrating the temporal evolution of serum lactate, amylase, and lipase levels during hospitalization.

Supplementary File 2: Summary table comparing clinical manifestations of dry, wet, and abdominal beriberi.

Supplementary File 3: Abdominal computed tomography image showing the duodenal hematoma.

Supplementary File 4: Diagram illustrating the mechanism of hyperlactatemia in thiamine deficiency.

Supplementary File 5: Clinical flowchart highlighting key features suggestive of thiamine deficiency in patients with abdominal pain and hyperlactatemia

Table 1 - Laboratory evolution demonstrating a decrease in serum lactate

	21/03/24	23/03/24	29/03/24	01/04/24	04/04/24	10/04/24	11/04/24 - thiamine onset	15/04/24	Reference values
Amylase	538	445	367	336	318	289			22-80 U/L
Lipase	767	496	501			423			5-31 U/L
Serum lactate					5,4	11,5	9,4	1,8	0,5-1,6 mmol/L

Table 2 - Comparison of Beriberi manifestations

**Type of Beriberi**	**Affected system**	**Symptoms**
**Dry Beriberi**	Peripheral and central nervous system	Numbness in the extremities, muscle weakness, tingling, loss of reflexes, muscle pain, difficulty walking. In severe cases, mental confusion, memory loss, and abnormal eye movements may occur.
**Wet Beriberi**	Cardiovascular	Tachycardia, dyspnea on exertion and lying down, lower limb edema, heart failure, congestion, hypotension
**Abdominal Beriberi**	Gastrointestinal	Acute and intense or insidious abdominal pain, nausea, vomiting, anorexia, hyperlactatemia.

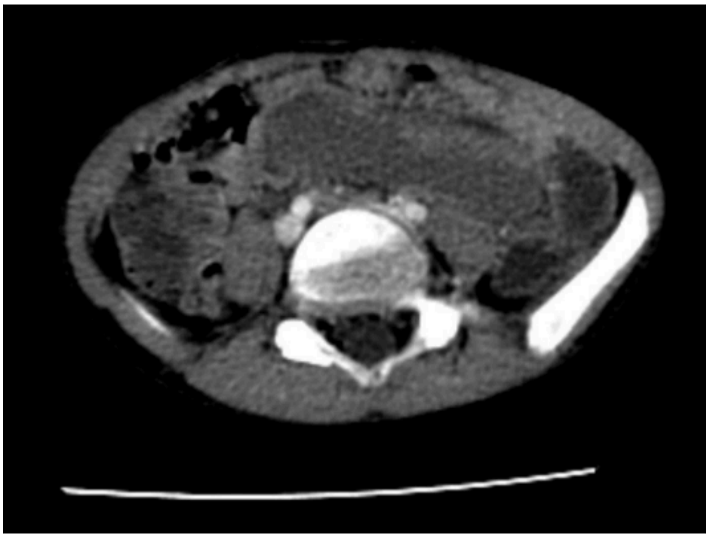

Figure 1: duodenal hematoma

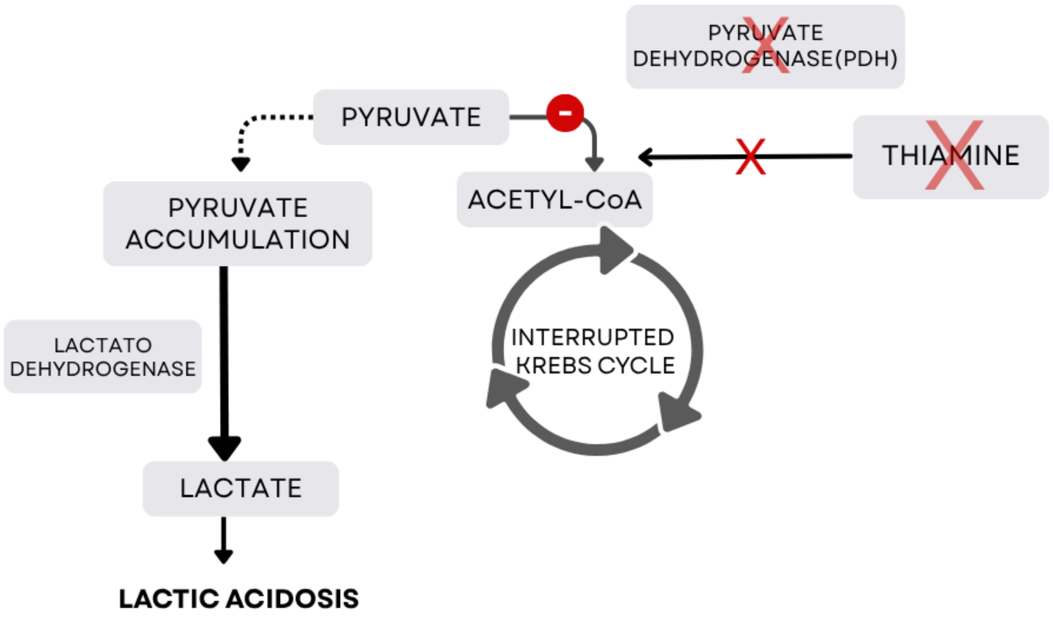

Figure 2 - Mechanism of lactate increase in thiamine deficiency

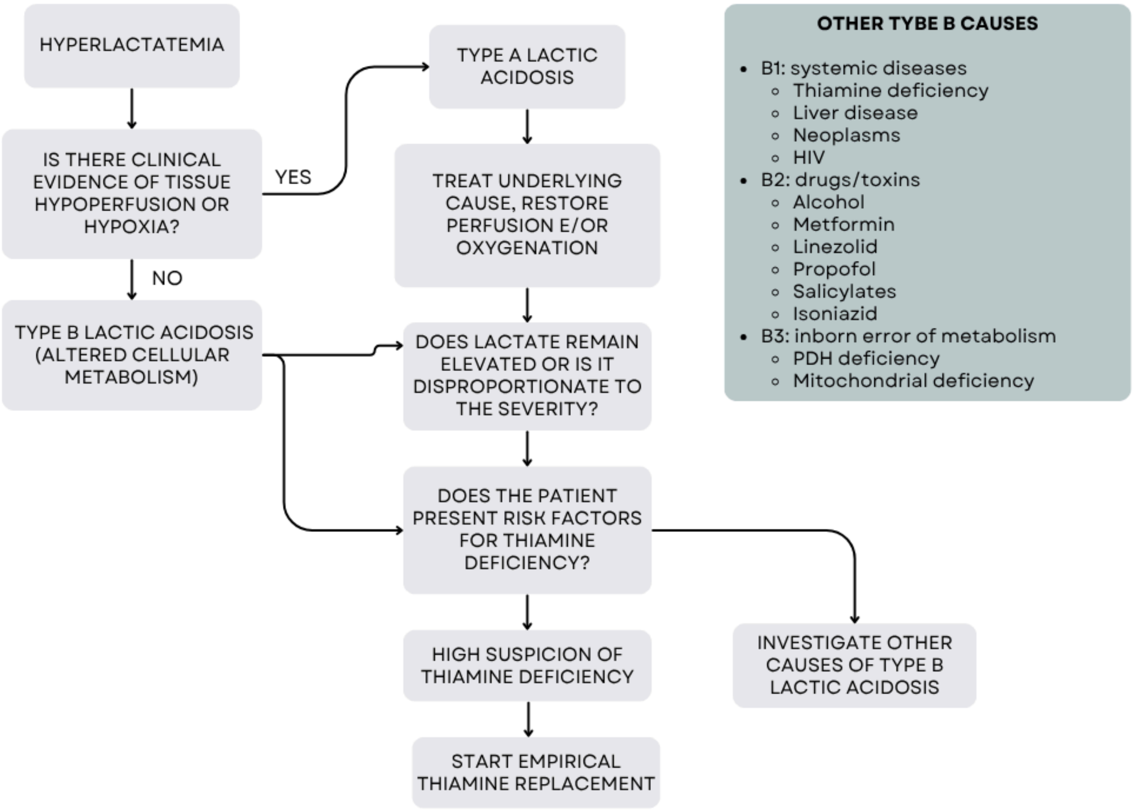

Figure 3 - Diagram to be suspicious of thiamine deficiency.
